# A Reliable, Label Free Quality Control Method for the Production of DNA Microarrays with Clinical Applications

**DOI:** 10.3390/polym13030340

**Published:** 2021-01-21

**Authors:** Elisa Chiodi, Francesco Damin, Laura Sola, Lucia Ferraro, Dario Brambilla, M. Selim Ünlü, Marcella Chiari

**Affiliations:** 1 Photonics Center, Department of Electrical Engineering, Boston University, Boston, MA 02215, USA; elich@bu.edu (E.C.); selim@bu.edu (M.S.Ü.); 2 Istituto di Scienze e Tecnologie Chimiche “G. Natta” SCITEC- Consiglio Nazionale delle Ricerche, 20131 Milano, Italy; lucia.ferraro@scitec.cnr.it (L.F.); dario.brambilla@scitec.cnr.it (D.B.); Marcella.chiari@scitec.cnr.it (M.C.)

**Keywords:** DNA microarray, spotting optimization, label-free detection

## Abstract

The manufacture of a very high-quality microarray support is essential for the adoption of this assay format in clinical routine. In fact, poorly surface-bound probes can affect the diagnostic sensitivity or, in worst cases, lead to false negative results. Here we report on a reliable and easy quality control method for the evaluation of spotted probe properties in a microarray test, based on the Interferometric Reflectance Imaging Sensor (IRIS) system, a high-resolution label free technique able to evaluate the variation of the mass bound to a surface. In particular, we demonstrated that the IRIS analysis of microarray chips immediately after probe immobilization can detect the absence of probes, which recognizably causes a lack of signal when performing a test, with clinical relevance, using fluorescence detection. Moreover, the use of the IRIS technique allowed also to determine the optimal concentration of the probe, that has to be immobilized on the surface, to maximize the target recognition, thus the signal, but to avoid crowding effects. Finally, through this preliminary quality inspection it is possible to highlight differences in the immobilization chemistries. In particular, we have compared NHS ester versus click chemistry reactions using two different surface coatings, demonstrating that, in the diagnostic case used as an example (colorectal cancer) a higher probe density does not reflect a higher binding signal, probably because of a crowding effect.

## 1. Introduction

Microarray technology has emerged as an essential tool for monitoring multiple biomolecular interactions between oligonucleotides, cDNA, proteins, or antibodies immobilized on the surface and their complementary targets in solution [1,2,3]. In different applications, like gene or protein expression profiling, analysis of point mutations, or immunodiagnostics, the quality of printed microarray slides is essential to determine the adoption of the technology in diagnostics [4]. Insufficient amounts of surface-bound probes can result in underestimating or even complete failure to detect the analyte of interest. Therefore, the realization of a uniform coating of a functional group, yielding spots of desired and reproducible density, shape, and size, has been subject to several studies [5,6]. The absence of consistent quality control exacerbates the effects resulting from the variability of print quality. Immobilization variability is not only related to physical parameters such as temperature and humidity. When multiple probes are spotted on the same surface, their attachment density depends on the probe concentration and stability of their functional groups [7]. While errors in preparing probe solutions might be operator dependent, their chemical integrity depends on the conditions to which the probe has been exposed, such as aging, number of freeze and thaw cycles, or solution contamination. The probe’s quality is difficult to assess due to the small volume used during spotting. Besides, defining criteria for quality control for a large number of probes might be a challenging task. Furthermore, during the development phase of a microarray assay, it is of utmost importance to establish the probe concentration that saturates the surface binding sites, which represents the density at which crowding effects diminish the mass of the target captured.

While methods that use fluorescent labels for visualizing printed arrays prior to hybridization have been presented [8,9], they are not the optimal choice for assay standardization or optimization. The ability to measure spot density using label-free techniques would provide valuable information on spot quality without altering standard microarray protocols. To account for the variability that primarily stems from variable DNA probe deposition and retention on the slide surface, a few label-free based methods have been devised to visualize printed slides prior to their experimental use [10,11]. However, their use in production quality control is limited by the fact that these methods often require a particular sensor configuration.

In this study, we present a new application for the Interferometric Reflectance Imaging Sensor (IRIS), a high-resolution, interferometry-based label-free imaging system [12] for the evaluation of spotted probe quality in a microarray test. In particular, the IRIS is an imaging-based technique, that allows for easy and robust assessment of ligand mass accumulation on a functionalized Si/SiO_2_ chip spotted with ligands (proteins, DNA, etc.) in an array format. The IRIS has developed over several iterations, from utilizing a laser light source, rotating ground glass and a large flow cell [13] to a more simplified and rugged multi-LED system, integrating sphere and a correspondingly simplified microfluidic chamber [12]. Here, the IRIS was utilized to generate pre-hybridization images of spotted oligonucleotide microarrays and measure real-time hybridization in a label-free manner, precisely quantifying the amount of mass bound to the surface. Spot intensity, size, level of saturation, and local background intensity are measured from DNA microarray images. This information is then used for the automated identification of missing (due to mechanical failure or sample depletion) or low-density spots and the estimation of the optimal concentration. The method also allows the user to compare different immobilization chemistries.

Our group has recently introduced a rapid and sensitive microarray-based assay for the multiplexed detection of minority mutations of oncogenes in tissue biopsies and plasma samples [14] of colorectal cancer. In this approach, single-strand DNA (ssDNA) hybridizes in solution with specific oligonucleotide reporters (dual-probe reporters) whose sequence consists of two domains. The 5’ domain is complementary to the gene of interest, such as *KRAS*, *NRAS*, or *BRAF* in their wild-type or mutated configuration, whereas the “barcode” 3’ domain recognizes oligonucleotide probes (capture probes) that are spotted at specific locations on the silicon chip. The ssDNA is captured on the array surface through hybridization with a barcode domain and then revealed by hybridization with a Cy3-labeled oligonucleotide (Universal-Cy3) complementary to a tag-primer added during PCR. Due to its multiplexable nature, the method allows the spotting of many different capture probes, whose sequence is designed to avoid interferences and cross-reactivity. In view of a large-scale adoption of this method, here we aimed at implementing non-destructive quality control of the spotting process and optimizing probe sequences to obtain an identical capture behavior in terms of kinetic and equilibrium constants. IRIS technology allowed to assess optimal surface concentration to avoid crowding effect, as well as to study the effect of probe orientation and surface density to achieve the highest signal at equilibrium. Optimizing spot quality increased pairwise correlation of post-hybridization spot intensity between replicate arrays, demonstrating that label-free spot quality captured the variability in the microarray data.

## 2. Materials and Methods

*N,N*-Dimethylacrylamide (DMA), 3-(trimethoxylsilyl)propyl methacrylate (MAPS), α,α′-Azoisobutyronitrile (AIBN), anhydrous tetrahydrofuran (THF), ammonium sulphate ((NH_4_)_2_SO_4_), phosphate buffered saline (PBS), ethanolamine, 3 were purchased from Sigma Aldrich (St. Louis, MO, USA). All solvents were used as received. *N*-acryloyloxysuccinimide and 3-azido-1-propylamine were synthesized as reported elsewhere [15,16]. MCP-4 copolymer was obtained by Lucidant Polymers Inc., Sunnyvale, CA, USA. All the oligonucleotides were synthesized by Metabion International AG (Steinkirchen, Germany). Their sequences are reported in [14]. These oligonucleotides were freeze-dried and re-suspended in deionized (DI) water at a final concentration of 100 μM before use.

Untreated silicon oxide chips with 100 nm thermal grown oxide (14 × 14 mm^2^) and with 110 nm thermal grown oxide (12.5 × 25 mm^2^) were supplied by SVM, Silicon Valley Microelectronics Inc. (Santa Clara, CA, USA). Chips were pretreated using a HARRICK Plasma Cleaner, PDC-002 (Ithaca, NY, USA) connected to an oxygen line. Spotting is perfomed using a SciFLEXARRAYER S12 (Scienion, Berlin, Germany).

InnoScan 710 (Innopsys, Carbonne, France) was used to scan the hybridized chips. Data intensities were extracted with the Mapix software and the data analysis was performed for each experiment. 

### 2.1. Surface Functionalization

Untreated silicon oxide chips, after a cleaning step (sonicating in acetone, rinse with methanol, deionized water, and dried with nitrogen), were activated by a treatment with oxygen plasma (15 min). This cleaning step is performed to eliminate the photoresist used in the fabrication of the chips, while the oxygen plasma activates the silanol groups on the surface. The activated substrates were immersed for 30 min in a copolymer solution (1% *w/v* in 0.45M (NH4)_2_SO_4_ water solution) and then rinsed with deionized water, dried under a nitrogen stream, and finally cured at 80 °C for 15 minutes.

Silicon chips were coated either with MCP-4 (Lucidant Polymers Inc., Sunnyvale, CA, USA) or Copoly Azide 10% copolymers. MCP-4 is a ter-copolymer of *N,N*-dimethylacrylamide (DMA), *N*-acryloyloxysuccinimide (NAS), and 3-(trimethoxysilyl)propyl methacrylate (MAPS) (89, 10, and 1% molar fraction, respectively), and it is synthesized by free radical polymerization according to the procedure described in [17]. MCP-4 is the variant of the original copoly-(DMA-NAS-MAPS) [18] a polymer typically used to coat glass, silicon, or other hydroxylated surfaces for microarray applications, in which the NAS molar fraction is increased from 2 to 10% to enhance its binding capacity.

**Figure 1:**
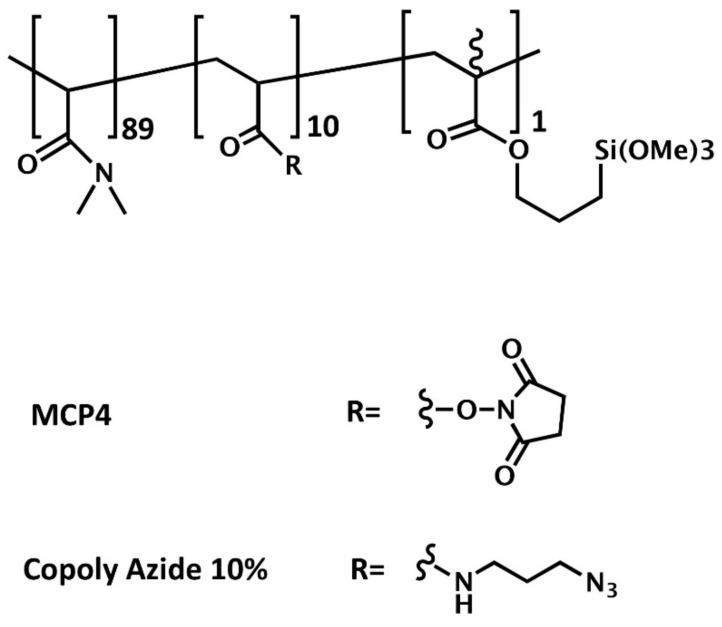
Structure of MCP-4 and Copoly Azide 10% copolymers. Both copolymers have a backbone of *N,N*-dimetheylacrilamide, but while MPC-4 contains *N*-acryloyloxysuccinimide as functional groups, which allows nucleophile substitution with amines, Copoly Azide 10% bears azide moieties for click chemistry reactions with alkyne or DBCO groups.

Copoly Azide 10% is an MCP-4 derivative where the activated ester of NAS is replaced by an azide moiety that allows azide-alkyne cycloaddition reactions, also known as “click” chemistry reactions [19]. Copoly Azide 10% was synthesized as described in [20,21]. The structure or the polymers is reported in Figure 1.

### 2.2. Microarray Preparation

In order to perform a preliminary control check, 33 different oligonucleotide capture probes, corresponding to 13 *KRAS* mutations, 13 *NRAS* mutations and 1 *BRAF* mutation, and to 6 wild-type sequences (details on sequences are reported in [14]), were printed, using a piezoelectric spotter equipped with an 80 µm nozzle, onto the surface of silicon chips with different silicon oxide thickness, depending on the detection technique used. In particular, chips with 110 nm thick SiO_2_ layer were used for IRIS, while 100 nm SiO_2_ layered chips were used for fluorescence detection. The capture probes, amino modified at the 5’ end, were diluted in printing buffer (150 mM sodium phosphate pH 8.5 containing 0.01% *w*/*v* sucrose monolaurate) at a final concentration of 10 μM and spotted, in duplicate, at 20° C in an atmosphere of 65% humidity. Depending on the different amount of bound mass observed in this first experiment, four capture probes were selected (in particular KRAS codon 12-13 wild type, KRAS G12C, KRAS G12D, and KRAS G13D mutations) for the optimization of the spotting concentration. These probes were immobilized on the chips, prepared as described previously, in quadruplicate at three different concentrations (10, 25, and 50 μM, respectively). The spotted chips were then placed in an uncovered storage box, laid in a sealed chamber, saturated with sodium chloride (40 g/100 mL H_2_O) and incubated overnight. After incubation, all residual reactive groups of the coating polymer were blocked by dipping the chips in a pre-warmed blocking solution (50 mM ethanolamine, 0.1 M Tris, pH 9.0) at 50 °C for 15 minutes, followed by rinsing twice in distilled H_2_O. Chips were washed in pre-warmed, post-coupling washing solution, 4X saline sodium citrate (SSC), 0.1% (*w*/*v*) sodium dodecyl sulfate (SDS), at 50 °C for 15 minutes, rinsed with distilled H_2_O, and dried by nitrogen stream.

### 2.3. Microarray Quality Control and Optical Setup

After the blocking step, label-free images of the dry blocked silicon chips (110 nm SiO_2_) for the quantification of the mass bound to the surface, were obtained with a real-time IRIS device, using a low-magnification IRIS setup. A detailed description of the instrument and its operation has been presented previously [22,23]. Briefly, the IRIS system works as a common path interferometer, where the collection path is rotated by 90° thanks to an elliptical turn mirror. The chip is illuminated by a four-color LED source (λ = 452, 518, 595, 632 nm) and the light reflected from the chip is collected by a CMOS Camera (FLIR GS3-U3-51S5M-C). The chip is imaged through a microfluidic chamber, built by attaching an antireflective (AR) coated glass slide to the chip through an adhesive gasket. The chamber is held in place by a clamping fixture, which keeps the chip under pressure and provides the contact points of the laser-drilled inlet and outlet holes with the microfluidic system that allows for liquid flow across the chip surface. The working principle of the IRIS is based on enhancing the signal generated by mass accumulation on a layered surface (a silicon chip with a 110 nm, thermally grown layer of silicon oxide on top) through constructive interference [24]. Essentially, the LED illuminates the chip surface, and the light, reflected from both the Si–SiO_2_ and SiO_2_–liquid interfaces, generates an interference pattern that can be tuned based on the oxide thickness and the illumination wavelength. The images were acquired through Micro-Manager software and analyzed in ImageJ to obtain the reflectance values. A custom-made MATLAB software was used to convert the raw signal data to mass density. The analysis is illustrated in detail in [25].

### 2.4. Dynamic Binding Measurement

For the dynamic-binding experiments, the IRIS chip was secured into a custom flow cell, as described in the previous section. The flow cell has a volume of 5 μL and is easily loaded into the IRIS instrument. Solutions were flowed through the flow cell and across the chip surface by using a peristaltic pump at an average flow rate of 200 μL/min. Before running the experiments, 2X SSC buffer was flowed for 15 min to condition the system.

The solutions of NRAS wild type, KRAS 12-13 wild-type, KRAS G12C, KRAS G12D, and KRAS G13D dual-probe oligonucleotides, each at a 50 nM concentration in 2X SSC buffer, were sequentially injected into the system and recirculated for 20 min at an average flow-rate of 200 µL/min. After the injection of each different dual-probe oligonucleotides, a washing step of 2X SSC buffer for 10 min was performed. Incubations and measurements took place at room temperature. Interferometric images of the binding to the corresponding capture probes were acquired during the whole process with a blue LED light (452 nm). There were four-color images, used to generate a look-up table for the conversion to mass density, acquired at the beginning of the experiment [25]. The binding curves were generated by converting the reflectance data to mass density (pg/mm^2^), then subtracting the background signal from the spot signal, and finally averaging the differential signal obtained from identical probe spots.

### 2.5. Optimal Spotting Concentration Characterization

**Figure 2:**
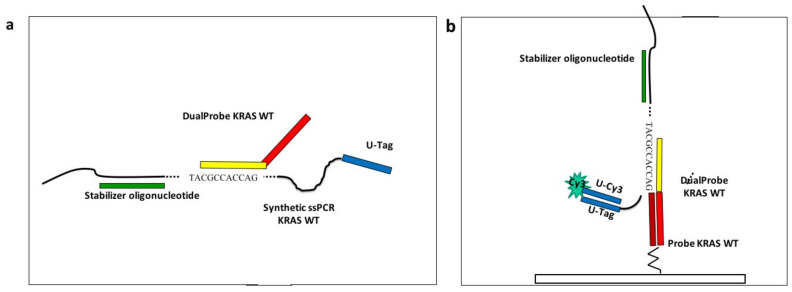
Scheme of the assay. (**a**) In the case of KRAS wild-type allele, the Stabilizer oligonucleotide (green rectangle) hybridizes in solution with the synthetic single strand PCR (ssPCR) to keep the synthetic ssPCR sequence stretched, then the Dual-Probe 5’ domain (yellow rectangle), complementary to the KRAS wild-type synthetic ssPCR, hybridizes in solution with its specific sequence inside the synthetic ssPCR. (**b**) The sequence in the 3’ domain of the Dual-Probe oligonucleotide (red rectangle) directs the synthetic ssPCR to its complementary capture probe on the array (dark red rectangle). The position in the array is revealed when the U-tag sequence at 5’-end of the synthetic ssPCR (blue rectangle) interacts with the complementary Cy3-labeled oligonucleotide, Universal-Cy3 (U-Cy3), added in the last step of the assay.

To simulate the assay described in a previous work [14], avoiding the complication of managing biological samples, and to study the optimal spotting concentration, we purchased four 84 mer long oligonucleotides (“synthetic single-strand PCR”) from Metabion International AG, (Steinkirchen, Germany) with the sequences corresponding to the fragment of the exon 2 of the KRAS gene, encompassing the mutation G12C, G12D, and G13D. This oligonucleotide mimics the single stranded amplicon, described previously [14], which was obtained after the thermal denaturation of the double strand PCR amplicon captured on magnetic microbeads. A detailed description of the assay protocol has been presented in [14] and briefly discussed here. The four synthetic ssPCRs were diluted at a final concentration of 50 nM in 2X SSC buffer in a volume of 1 mL. The four solutions were subjected to a thermal step (95 °C for 5 min), to avoid the presence of secondary structures. At the end of the thermal stage, the stabilizer (50 nM), an oligonucleotide necessary to keep the oligonucleotide sequence stretched, was added to each tube and incubated for 10 minutes at room temperature. Finally, in order to obtain the binding of the synthetic-ssPCRs to the corresponding capture probes on the chips (110 nm SiO_2_), the dual-probes for KRAS wild-type and G12C, G12D, and G13D mutations were added to the corresponding tubes, at a final concentration of 50nM. The incubation lasted for 35 minutes with gentle rotation in a stepwise gradient of temperature ranging from 42 to 29 °C. After the liquid allele-specific hybridization, the universal oligonucleotide labelled with Cy3 (Universal-Cy3) was added to the synthetic ssPCR-dual probes solutions to a concentration of 50 nM. A scheme of the experiment is reported in Figure 2.

### 2.6. Fluorescence Detection Assay

In parallel to the dynamic binding assay, a fluorescence detection assay was performed by hybridizing 20 μL of the samples, prepared as described above, on four different functionalized Si/100 nm-SiO_2_ chips (one chip for each sample).

The solutions were spread onto the silicon substrates and cover slips were placed on the spotted area. The chips were incubated at room temperature for 15 min in a humid hybridization chamber. Finally, the silicon chips were removed from the hybridization chamber and soaked briefly in 4X SSC buffer to remove the cover slips, then dipped, in sequence, in a solution 0.2X SSC and 0.1X SSC for 1 min at room temperature, dried with a nitrogen flow and scanned using a InnoScan 710 (Innopsys, Carbonne, France), applying a green laser (λ_ex_ 532 nm) for the Cy3 dye. The photomultiplier (PMT) tube gain and the laser power changed between different experiments. 16-bit TIFF images were analyzed at 5 µm resolution. Data intensities were extracted with the Mapix software and the data analysis was performed for each experiment.

## 3. Results and Discussion

In a previous work [14], we have designed a microarray-based assay for the multiplexed detection of minority mutations of oncogenes (*KRAS*, *NRAS* and *BRAF*) with relevant diagnostic implications in tissue biopsies and plasma samples in metastatic colorectal cancer patients. However, in such applications, and also in view of a possible industrial exploitation of the assay, a quality control of the spotted array is fundamental to avoid diagnostic failure. In fact, inadequate quantity or poorly bound probes may result in unyielding target recognition, invalidating the accuracy of the entire assay and leading to false negative results. For this purpose, we have introduced a methodology for checking the quality of the spots on the microarray chips. In particular, we have combined a dynamic label-free binding assay with a fluorescence test to investigate the optimal concentration of each bound probe, which results in an optimal fluorescence detection, in terms of specificity, sensitivity and accuracy. The label-free dynamic binding assay provides, in fact, a piece of information which would be impossible to obtain otherwise: the real amount of the initially immobilized mass, as well as the final, hybridized quantity. Knowing the initial surface density allows to estimate the spotting yield; similarly, monitoring the binding reaction and assessing the final hybridized quantity, enables a precise quantification of the activity of the immobilized probes, and an investigation on the potential presence of crowding effects.

**Figure 3:**
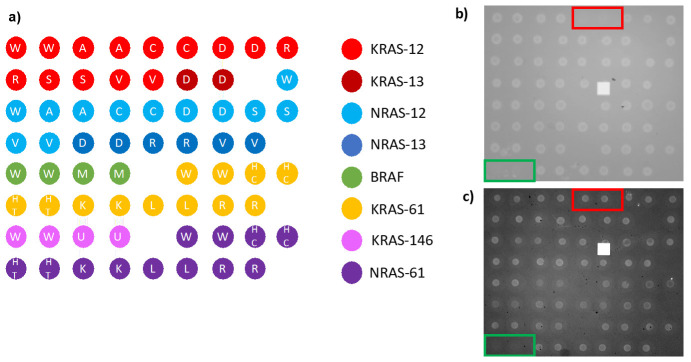
Scheme and images of the multiplexed chip utilized for IRIS quality control assay. (**a**) Spotting scheme of the 33 oncogene capture probes, where different colors indicate a different codon or gene. (**b**) IRIS image of a chip where KRAS-12 C (red rectangle) and NRAS-61-ht (green rectangle) were absent due to poor immobilization. (**c**) IRIS image of a new chip where immobilization for KRAS-12-C was optimized (red rectangle).

To this extent, two different types of experiments were carried out. In one case, the chip was spotted with all the mutations of KRAS, NRAS, and BRAF oncogenes (Figure 3a), and five complementary sequences (Dual probe reporters) were flowed across the active, spotted surface. 

**Figure 4:**
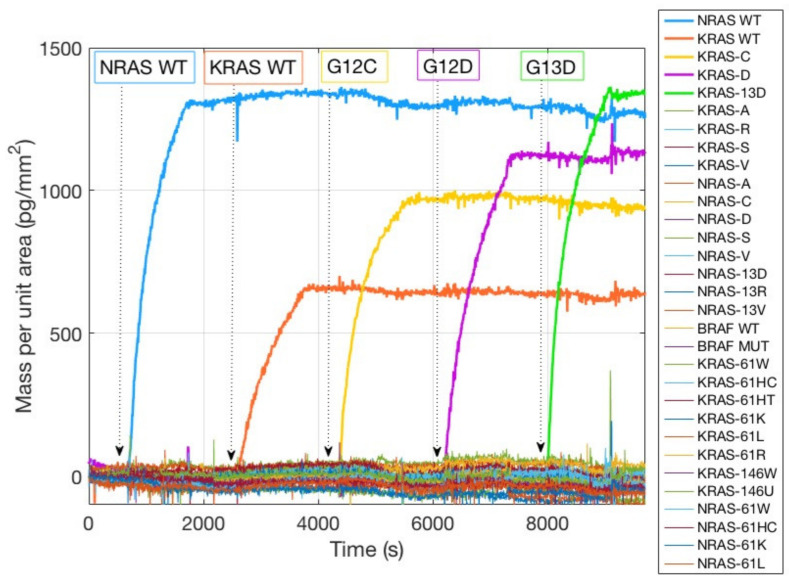
Binding curves obtained with the Interferometric Reflectance Imaging Sensor (IRIS) system for the specific capture of DNA target sequences. The five samples at 50 nM (NRAS WT, KRAS WT, G12C, G12D, G13D) were injected sequentially into the fluidic chamber and bound stably to their complementary probe (NRAS WT, KRAS WT, KRAS 12C, KRAS 12D, KRAS 13D, respectively, thick lines). No non-specific binding was detected on any of the other probes (thin lines).

Figure 3 shows the spotting scheme of the 33 oncogene capture probes corresponding to the KRAS, NRAS, and BRAF oncogenes, together with the initially immobilized mass for all the dry spotted probes. The surface density is fairly consistent across most spots (Figure 3b,c), confirming the ability of MCP-4 polymer to stably immobilize amine-modified probes. However, two probes were not immobilized correctly, in particular KRAS 12C and NRAS 61HT (Figure 3b red and green rectangles). There could be more than one explanation for the immobilization failure: aging of the capture probe can be a motivation, but most probably the amine tail on the DNA strand was deteriorated, and the coupling reaction did not perform as expected. The replacement of the old probe with a fresh one, resulted in a better immobilization in terms of probe quantity (2 ng/mm^2^ vs. 0.4 ng/mm^2^, see Figure S1 Supporting Information), as it can be noticed from Figure 3c, red rectangle. Lack of immobilization as that evidenced in Figure 3b would inevitably lead to a negative result in the detection of that specific mutation since the recognition event cannot occur, thus affecting and invalidating the diagnostic capability of the assay. On the contrary, thanks to this label-free quality control method, the user can quickly establish if there is a problem connected to the immobilization step, gaining a piece of information that is impossible to obtain with fluorescence. The real-time hybridization experiment was performed in order to validate the specificity of the probes by introducing into the fluidic cell oligonucleotide sequences (Dual probe reporters) complementary to five of the spotted probes. As it can be observed in Figure 4, all the target sequences are highly specific since they only bind to their complementary on the surface. Non-specific binding was not detected on any of the other spotted mutations.

This high specificity was expected since the target sequences were synthetically produced to match the probes. However, experimental verification of the absence of cross reactivity between the probes is advisable when working with real clinical samples.

A second investigation was performed in order to determine the best spotting concentration to preserve the reactivity of the probes. At the same time, the difference in terms of immobilization strategy was evaluated. To this extent, the immobilization through NHS active ester (obtained on MCP-4 coated surfaces) was compared to that achieved on a surface coated with an azide bearing copolymer, which allows a click-chemistry based immobilization through a 1,3-dipolar cycloaddiction reaction [20]. In this case, all the capture probes were modified with a DBCO group instead of an amine; the coating and spotting procedure were, however, identical to those reported in Section 2.2.

Covalent immobilization of biomolecules through reaction with NHS ester is widely exploited [26,27] because NHS active ester is a functional group highly reactive towards nucleophiles such as amine, a group naturally present in proteins and peptides that can be also easily inserted in oligonucleotides. The nucleophile substitution does not require harsh conditions and the resulting amide bond is very stable. However, the use of active esters has some drawbacks, mainly because of their instability on water. On the other hand, the so called click chemistry reactions overcome these issues offering quantitative yields, controlled orthogonal and chemoselective probe immobilization, and insensitivity to pH. In this work, we have exploited the so called Strain Promoted Alkyne-Azide Cycloaddition (SPAAC), a bioorthogonal reaction utilizing a pair of reagents, cyclooctynes (present in the DBCO group, inserted at the 5’ end of the oligonucleotide strand) and azides (inserted in the polymer chains by post-polymerization modification) that exclusively and efficiently react with each other while remain inert to naturally occurring functional groups such as amines.

The two different surface chemistries have an impact also on probe orientation. The orientation of the probe on the surface mainly depends on the probes itself, the surface, and the ionic strength of the environment. In this work, the probes immobilized on the surface of the chip are single stranded DNA oligonucleotides, whose single binding site to the surface is the amine moiety introduced at 5’ end during their synthesis. Furthermore, DNA is well-known to have a net negative charge. In Spuhler P. et al. [28] we have demonstrated that the combination of DNA negative charges, together with the remaining charges on the coated surface (with this particular ionic strength environment) are enough to generate significant elevation of the probe. However, probes different from DNA (such as protein or peptide) have more functionalities in their structure which may interact with the surface, leading to non-specific binding, thus causing loss of activity and reduced probe conformational flexibility. To this extent, we have introduced click chemistry immobilization which generates accurate probe orientation on the surface, greatly affecting the analytical outcome of the array [29].

**Figure 5:**
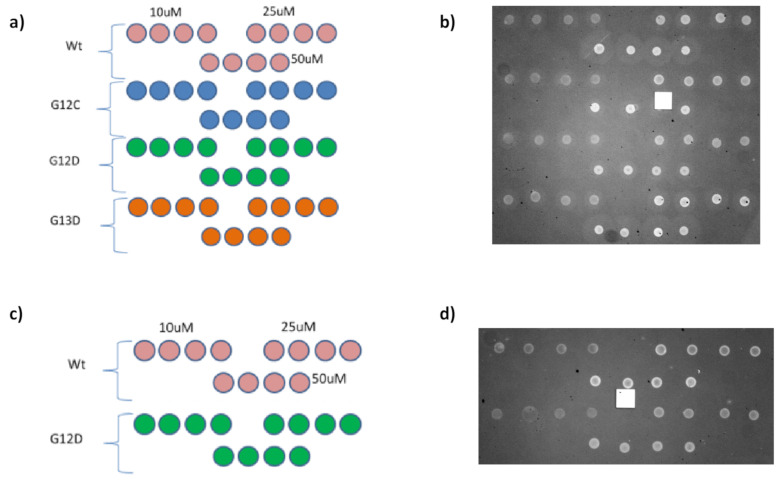
Spotting scheme on (**a**) MCP-4 and (**c**) Copoly Azide 10% coated surfaces and IRIS images of the (**b**) MCP-4 and (**d**) Copoly azide 10% chips, taken immediately after spotting.

In order to measure the effect of surface density on probe reactivity, three different concentrations of a set of distinct KRAS mutations were spotted on MCP-4 or Copoly azide 10% functionalized IRIS chips. Overall, four mutations were immobilized on MCP-4-coated chips, while two were spotted on azide-modified substrates. The spotting scheme for these experiments is reported in Figure 5, together with IRIS images of the dry chips, taken before starting the experiment.

**Figure 6:**
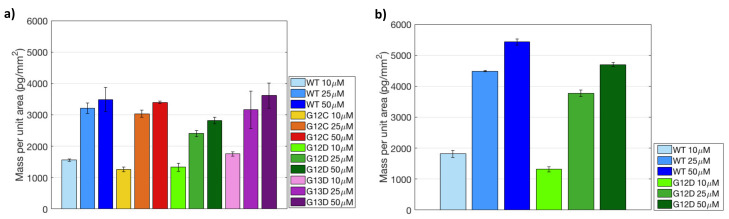
Immobilized mass of the probes spotted on (**a**) MCP-4 and (**b**) Copoly Azide 10% coated chips, obtained by analyzing the IRIS images of Figure 5.

The initially immobilized mass for both surface chemistries (MCP-4 and Copoly Azide 10%) is shown in the histograms of Figure 6.

**Figure 7:**
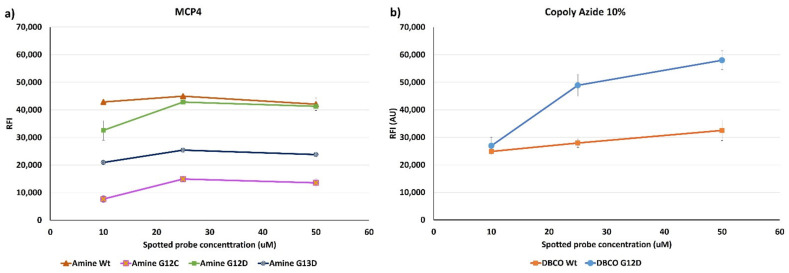
Fluorescence signal intensities obtained after hybridization of the spotted probe with a synthetic ssPCR. Fluorescence detection has been obtained using a Cy3-labelled oligonucleotide (U-Cy3) complementary to the U-tag sequence at 5’-end of the synthetic ssPCR. The same experiment was performed on (**a**) MCP-4 coated chips, onto which amine modified oligonucleotide were immobilized, and on (**b**) Copoly Azide 10% Coated chips; in this case DBCO modified probes were used. Fluorescence was detected using a confocal laser scanner, setting the power to low and the gain to 1%.

Comparing the two histograms, it can be noticed that increasing the spotting concentration on MCP-4 coated chips leads to saturation of the available binding sites. Indeed, the mass immobilized at 25 μM is fairly similar to the one immobilized at 50 μM, for all probes. Conversely, on Copoly Azide 10% coated surface, a linear increase in mass density was observed with the increase in concentration. These results seem to imply that Copoly Azide 10% is more efficient in terms of immobilization, which was also confirmed by fluorescence measurements. Figure 7 shows the fluorescence signals obtained after hybridization with a Cy3-labelled oligonucleotide (U-Cy3) complementary to the U-tag sequence at 5’-end of the synthetic ssPCR (see Figure 2). In particular, on chips coated with Copoly Azide 10%, a linear correlation between the spotted probe concentration and the fluorescence signal can be observed, meaning that Copoly Azide 10% can allocate a higher probe density which remains active. On the contrary, on MCP-4 coated surfaces, increasing the spotted probe concentration does not result in an increase in fluorescence signal, which reaches a plateau around 25 μM of spotted probe.

**Figure 8:**
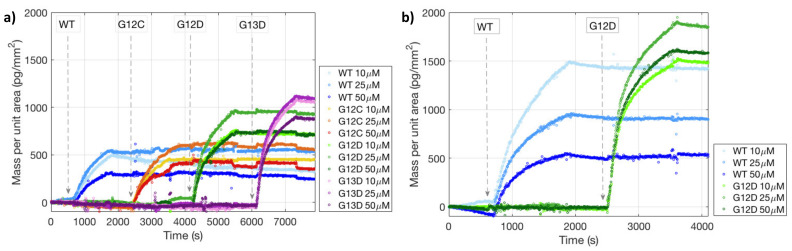
Binding curves obtained with the IRIS system for complementary targets binding to the respective probes at different concentration on a (**a**) MCP-4 (**b**) Copoly Azide 10% coated surface.

The performance of the fluorescence assay was then compared with a dynamic binding assay performed on both MCP-4 and Copoly Azide 10% coated chips. In particular, the dynamic binding assay, performed in the IRIS instrument produced the binding curves reported in Figure 8.

Interestingly, in most cases, 25 μM was the spotting concentration that yielded the highest binding signal, and, therefore, seemed to be the optimal concentration able to preserve probe activity. A possible reason is that increasing the concentration above 25 μM could cause crowding effects, while lowering it below that level could possibly prevent the maximization of the binding efficiency. Consistently with fluorescence data, binding signal was much higher on azide-functionalized chips, which, overall, seems to suggest a better and more ordered distribution of the probe sequences on the surface.

The correlation between fluorescence and label free results further highlights the urgency of a proper immobilization strategy, therefore the significance of a quality control method to avoid errors and oversights in data interpretation.

## 4. Conclusions

We have introduced a method that allows an easy validation of the quality of DNA microarrays, both for specificity and activity of the probes. In particular, the analysis by IRIS of microarray chips immediately after probe immobilization demonstrated that the absence of immobilized probe might be the reason of lack of signal when performing fluorescence detection. This quality control is fundamental in developing DNA mutation-based microarray assays with diagnostic applications, in order to maximize the test accuracy and reduce the false negative results. Such a method would be of crucial support in an industrial setting. Furthermore, we have also optimized the immobilized probe concentration, demonstrating that this parameter has a role on the final assay performance. Differences on the immobilization chemistry (NHS ester vs. click chemistry) have been highlighted as well: in particular, on azide functionalized surfaces higher concentration of probes can be accommodated because of a higher binding capacity of the surface. However, a higher probe density does not reflect a higher binding signal, probably because of a crowding effect which does not allow proper target pairing.

## Data Availability

The data presented in this study are available on request from the corresponding author.

## Supplementary Materials

The following are available online at https://www.mdpi.com/2073-4360/13/3/340/s1, Figure S1: Mass per unit area of the immobilized oligonucleotide probes calculated with the IRIS setup.
