# Supplementary material for: A Reliable, Label Free Quality Control Method for the Production of DNA Microarrays with Clinical Applications
Source: Polymers (Basel). 2021 Jan 21;13(3):340. doi: 10.3390/polym13030340 (PMC7865641; doi:10.3390/polym13030340)
Supplement: Supplementary file 1 [file polymers-13-00340-s001.pdf]

Supplementary Materials

A Reliable, Label Free Quality Control Method for the Production of DNA Microarrays with Clinical Applications

Elisa Chiodi [1], Francesco Damin [2,*], Laura Sola [2,*], Lucia Ferraro [2], Dario Brambilla [2], M. Selim Ünlü [1] and Marcella Chiari [2]

[1] Photonics Center, Department of Electrical Engineering, Boston University, Boston, MA 02215, USA; elich@bu.edu (E.C.); selim@bu.edu (M.S.U.)

[2] Istituto di Scienze e Tecnologie Chimiche "G. Natta" SCITEC- Consiglio Nazionale delle Ricerche, 20131 Milano, Italy; lucia.ferraro@scitec.cnr.it (L.F.); dario.brambilla@scitec.cnr.it (D.B.); Marcella.chiari@scitec.cnr.it (M.C.)

* Correspondence: francesco.damin@scitec.cnr.it (F.D.); laura.sola@scitec.cnr.it (L.S.); Tel.: +39 02 28500042 (F.D.&L.S.)

The preliminary check of the dry spotted chip allows to determine whether the probe has been properly immobilized or not, avoiding a false negative result when performing the fluorescence detection of a microarray test. These histograms (Figure S1) report the mass per unit of the 33 oligonucleotide capture probe corresponding to the KRAS, NRAS, and BRAF genes, calculated after analysis with the IRIS setup of the spotted chip.

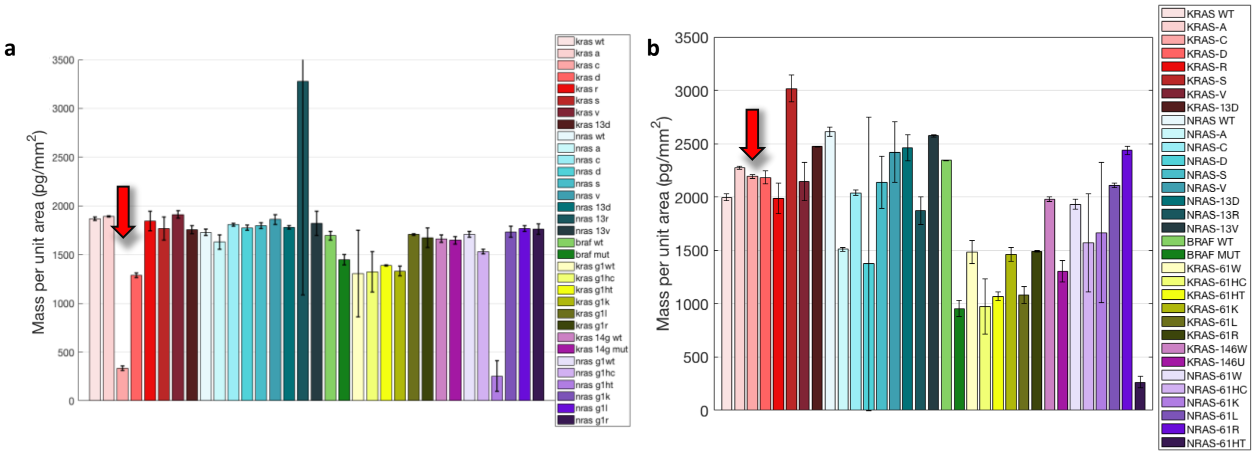

Figure S1. Mass per unit area of the immobilized oligonucleotide probes calculated with the IRIS setup.

As can be noticed, in Figure S1a, the mass calculated for the probe KRAS 12 C (red arrow) is 0.4 ng/mm². Such a low amount of immobilized probe would give a negligible signal in fluorescence detection, which would be classified as a not occurring interaction, thus a false negative when performing a diagnostic assay.

By changing the oligonucleotide probe with a fresh batch, the immobilized mass was then calculated to be 2 ng/mm² (Figure S1b, red arrow).

The IRIS set up offers an easy and straightforward method to perform a quality control of the spotted dry chips to avoid evaluation errors when performing a fluorescence assay.
